# Mesenchymal Stem Cell Sheet Promotes Functional Recovery and Palliates Neuropathic Pain in a Subacute Spinal Cord Injury Model

**DOI:** 10.1155/2021/9964877

**Published:** 2021-07-09

**Authors:** Kazuyoshi Yamazaki, Masahito Kawabori, Toshitaka Seki, Soichiro Takamiya, Kotaro Konno, Masahiko Watanabe, Kiyohiro Houkin, Miki Fujimura

**Affiliations:** ^1^Department of Neurosurgery, Graduate School of Medicine, Hokkaido University, Japan; ^2^Department of Anatomy and Embryology, Graduate School of Medicine, Hokkaido University, Japan

## Abstract

Stem cell therapy has been shown to reverse the sequelae of spinal cord injury (SCI). Although the ideal treatment route remains unknown, providing a large number of stem cells to the injured site using less invasive techniques is critical to achieving maximal recovery. This study was conducted to determine whether administration of bone marrow stem cell (BMSC) sheet made on its own without a scaffold is superior to intramedullary cell transplantation in a rat subacute SCI model. Adult female Sprague-Dawley rats were subjected to SCI by 30 g clip compression at the level of Th6 and Th7 and were administered BMSC cell sheet (7 × 10^4^ cells, subdural), cell suspension (7 × 10^4^ cells, intramedullary), or control seven days after the injury. Motor and sensory assessments, as well as histological evaluation, were performed to determine the efficacy of the different cell transplantation procedures. While both the cell sheet and cell intramedullary injection groups showed significant motor recovery compared to the control group, the cell sheet group showed better results. Furthermore, the cell sheet group displayed a significant sensory recovery compared to the other groups. A histological evaluation revealed that the cell sheet group showed smaller injury lesion volume, less inflammation, and gliosis compared to other groups. Sensory-related fibers of *μ*-opioid receptors (MOR, interneuron) and hydroxytryptamine transporters (HTT, descending pain inhibitory pathway), located around the dorsal horn of the spinal cord at the caudal side of the SCI, were preserved only in the cell sheet group. Stem cells could also be found inside the peri-injured spinal cord in the cell sheet group. BMSC cell sheets were able to promote functional recovery and palliate neuropathic pain more effectively than intramedullary injections, thus serving as a good treatment option for SCI.

## 1. Introduction

More than one million patients in the world suffer from paralysis caused by spinal cord injury (SCI) [1]. In addition to paraparesis, SCI patients often complain of progressive neuropathic pain below the level of injury [2]. It is well established that pain strongly influences the deterioration of physical, mental, and social functions and causes restriction of rehabilitation by interfering with daily life and sleep [3–5]. Therefore, the development of effective treatment methods can improve not only motor dysfunction but also sensory impairment because of SCI.

Stem cell therapy, which regenerates neurological networks or ameliorates neural damage, is expected to be a promising candidate for SCI, and numerous clinical investigations have been launched, with various results [6, 7]. Although the cell transplantation methods, such as the type of cells, doses, transplantation routes, and timing, vary between trials and there is no consolidated consensus on the safest and most effective treatment strategy, intramedullary injection remains the most popular route of transplantation. This is because intramedullary transplantation has shown superiority in delivering the highest number of cells and providing greater functional recovery over intravascular (arterial or venous) transplantation in animal experiments [8, 9]. However, the major concern with intraspinal injection of stem cells is that it possesses the risk of mechanical injury during the injection procedure, as cells need to be injected by needles which can damage the spinal cord or blood vessels. To overcome this issue, stem cells with scaffolds have been intensively investigated [10–14]. Importantly, if the scaffold containing stem cells successfully attaches to the spinal cord surface, it can supply a sufficient number of cells to the injured area while avoiding additional injury. However, scaffolds have been reported to be limited in that cells encapsulated in the scaffold may show limited cell survival and differentiation, integrate inadequately into the host tissue, and aggravate local inflammation [15–19]. Recent advances in cell and tissue engineering have achieved the development of stem cell sheets, which require no biomaterials as scaffolds as they are created solely from stem cells and clinical trials on the application of these sheets for other diseases have been launched [20–24]. However, data supporting the use of stem cell sheets for SCI are not fully elucidated [25]. Therefore, the aim of this study was to examine the therapeutic efficacy of subdural administration of a bone marrow mesenchymal stromal cell (BMSC) sheet in an SCI compression model, especially focusing on the alleviation of sensory impairment.

## 2. Materials and Methods

### 2.1. Experimental Ethics

All experiment protocols were approved by the Hokkaido University Graduate School of Medicine Animal Studies Ethical Committee (reference number 17-0065). All procedures were performed by following the institutional and governmental guidelines (Guidelines for Proper Conduct of Animal Experiments by the Science Council of Japan).

### 2.2. Isolation and Culture of BMSCs

The BMSCs were isolated from 10-week-old, green fluorescent protein (GFP) expressing transgenic Sprague-Dawley (SD) rats (Japan SLC Co., Ltd., Shizuoka, Japan) as previously described. [26, 27] The cells were passaged three times before being used in the experiments.

### 2.3. Preparation of BMSC Sheets

**Figure 1:**
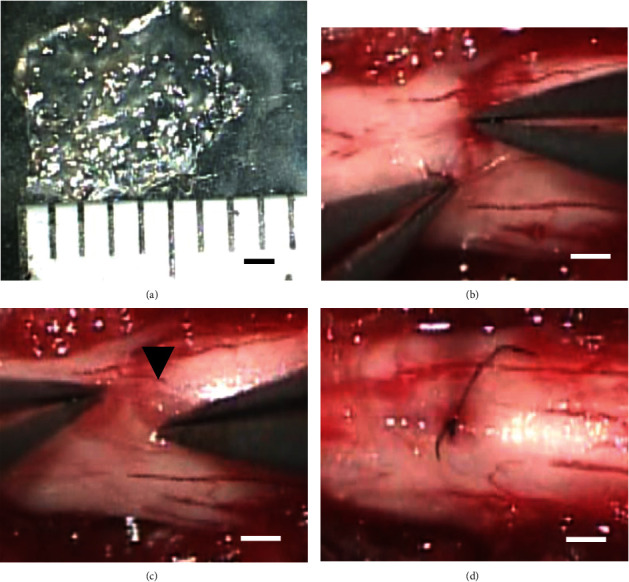
BMSC cell sheet and transplant image. (a) BMSC cell sheet detached from DISH and trimmed before transplantation. Scale bar represents 1 mm. (b) Incision of the dorsal dura mater at the spinal cord injury site. Scale bar represents 500 *μ*m. (c) Image of implanting BMSC cell sheet under the dura mater of the injury site. Scale bar represents 500 *μ*m; arrow points to the BMSC cell sheet. (d) Image after suturing the dura mater with 10-0 nylon thread. Scale bar represents 500 *μ*m.

The BMSC cell sheet was created as previously described with some modifications [28]. Briefly, 8.8 cm^2^ culture dishes with a temperature-responsive polymer (poly-N-isopropyl-acrylamide) coating (CellSeed Inc., Tokyo, Japan) were used to prepare the BMSC sheets following the manufacturer's instructions. BMSCs were seeded onto the dishes of 5 × 10^5^ cells/dish density with 3 ml of minimum essential medium (MEM) *α* (Invitrogen, 32571-036, USA) containing 10% fetal bovine serum (FBS), 0.2% 100 unit/ml penicillin G, and 82 *μ*g/ml L-ascorbic acid (Wako Pure Chemical Industries, No. 013–19641, Osaka, Japan). The cells on the dishes were incubated in incubation chamber (5% CO_2_ and 95% air at 37°C), and the medium was changed two to three times per week until the cells reached confluence. After 7 days of culture, the dish with confluent BMSCs was incubated at 20°C, and within 10 to 20 min, the cells were able to detach and float spontaneously up into the medium as a single-cell sheet (Figure 1(a)). For counting the cell number in each sheet, the BMSC sheet (*n* = 5) was digested using 0.05% trypsin (Gibco, Tokyo, Japan), and 1.2 ± 0.2 × 10^6^ cells were obtained from each cell sheet. For aligning the number of cells with that in the intramedullary injection group, the sheet was cut into 7 × 7 mm squares and adjusted so that approximately 7.0 × 10^4^ cells would be transplanted.

### 2.4. Experimental Animals

A total of 60 wild-type eight to ten-week-old female SD rats (250-300 g) (CLEA Japan, Inc., Tokyo, Japan) were used in these experiments. Females were used because of urination management issues that arise after SCI. Animals were allowed free access to water and food and kept in a controlled environment (50% humidity, 25°C, and 12-hour light-dark cycle).

### 2.5. SCI Model

The method for inducing SCI has been previously reported. [29–31] Briefly, one-minute compression by modified aneurysm clip (Mizuho, Tokyo, Japan) was used to induce thoracic SCI. The clip was designed to have 30 g closing force, and the blade was smoothened for applying equal pressure to the spinal cord. Anesthesia was induced with isoflurane at a starting concentration of 4.0% and maintained at 2.0%, in 30% O_2_ gas and 70% N_2_O, through a mask. After anesthesia, laminectomy was performed in the prone position at the vertebral segments Th6 and Th7. Then, the clip was extradurally inserted to pinch the spinal cord at the Th6/Th7 level for one minute. Rats that presented low motor deficit (Basso, Beattie, and Bresnahan (BBB) score of more than 2 points 24 hours after insult) were excluded from subsequent experiments. The manual compression of rats' bladders was performed for urination 3 times a day until spontaneously bladder control was obtained.

### 2.6. Cell Transplantation

Cell transplantation was performed 7 days after SCI. All experimental animals were randomly divided into one of the following groups: the subdural transplantation of BMSC cell sheet group (cell sheet group, *n* = 20), BMSC cell suspension intramedullary injection group (intramedullary injection group, *n* = 20), and control group (*n* = 20). During the transplantation procedures, the rats were anesthetized as mentioned above. In the cell sheet group, the dura mater at the site of the SCI was incised approximately 2 mm longitudinally (Figure 1(b)), and the cell sheet slowly slid under the dura mater (Figure 1(c)). The dura mater was then sutured with a 10-0 nylon thread (Figure 1(d)). In the intramedullary injection group, 7.0 × 10^4^ cells suspended in saline were injected directly into the spinal cord 1 mm rostral from the injured site using a stereotactic apparatus (Model DKI-900; David Kopf Instruments, Tujunga, CA) [32, 33]. In the control group, only the dura incision was performed in the same manner as that in the cell sheet transplanted group, and the dura was sutured without transplanting the cells.

### 2.7. Neurological Score

Two blinded independent observers carried out all assessments on days 1, 7, 14, 21, 28, 35, 42, and 49 post-SCI injury as previously described. [31–33] The BBB hind limb locomotor test was evaluated to determine motor deficits by observing movement for 5 minutes in an open field. The von Frey monofilament test (Touch Test Sensory Evaluator Kit, Stoelting Co., Wood Dale, IL) was conducted to evaluate sensory impairment for hyperalgesia. Briefly, the rats were placed on a floor and von Frey hairs were used to evaluate the threshold of neuropathic pain in response to mechanical insult ranging from 0.008 to 300 g on both the hind paws and dorsal trunk surface, mainly the tail base. Impacts that induced a consistent rapid avoidance behavior (a frightened move elicited by pain) and vocalization were considered the withdrawal thresholds. The smallest impact of hind paws or trunk which caused the withdrawal action, in most cases the trunk, was considered as its threshold. The rats with neuropathic pain react even to this very slight sensational feeling, whereas normal rats often ignore and do not move with such low-pressure touches or slowly move forward with high-pressure stimuli [34]. Animals died during the study periods were excluded from the analysis.

### 2.8. Evaluation of SCI Lesion Volume

Hematoxylin-eosin staining for staining neurons and Luxol fast blue staining (LFB) for evaluating myelin were used to estimate the SCI lesion volume, 49 days after SCI, as previously reported [29, 31]. At the time of sacrifice, the rats were anesthetized deeply with 4.0–5.0% isoflurane in N_2_O/O_2_ (70 : 30) to avoid pain. They were transcardially perfused with saline (50 ml), followed by paraformaldehyde (50 ml of 4%). The spinal cord (Th2–Th11) was removed and fixed in paraformaldehyde at 4°C for 3 days and soaked in the decalcification liquid made of PBS(-) containing 50 mg/ml EDTA/2Na (349-01865, Dojindo, Japan) and 50 mg/ml EDTA/4Na (349-01885, Dojindo, Japan) for 14 days. Then, the blocks were embedded in paraffin and sagittal sections (10 *μ*m thick) or transverse sections (4 *μ*m thick) were mounted onto slides for immunostaining. The lesion length and volume were evaluated as previously described [29, 31, 35]. Briefly, sagittal sections of the spinal cord were used, and the distance between the epicenter of the injured spinal cord and the most caudal and rostral point was determined by the discontinuation of LFB staining semiautomatically analyzed by BZ-X Analyzer (Keyence, Osaka, Japan). The lesion volume was analyzed by the following formula: lesion volume = *πD*^2^ (H1 + H2)/6 (H1 is the length between the epicenter and the rostral end, H2 is the length between the epicenter and the caudal end, and *D* is the diameter of the epicenter).

### 2.9. Immunohistochemistry

Local Inflammation was evaluated 49 days after SCI (*n* = 5 each) using sagittal sections of the spinal cord. Neuroinflammation was examined based on Iba1 and CD68 secretions, as previously reported [31, 37, 38]. Anti-Iba1 antibody (1 : 1500 at room temperature for 1 hour, 019-19741, Wako, Japan) and anti-CD68 antibody (1 : 1000 at room temperature for 1 hour, MCA341GA, Bio-Rad) were used for immunohistochemistry. Following the primary antibody reaction, Histofine® Simple Stain Rat MAX PO (Nichirei Bioscience Inc.) was adopted for 1 hour. Then, the DAB Substitute Kit (Nichirei Bioscience Inc.) was used following the manufacturers' instructions. Images were obtained from the peridamaged lesion (5 mm rostral and caudal from the epicenter of the damaged lesion). A total of five nonoverlapping areas were selected, and the cells showing positive signals were semiquantitatively counted using an automated cell counting software (BZ-X Analyzer, Keyence, Osaka, Japan). Posttraumatic glial scar formation was analyzed using sagittal sections; the glial fibrillary acidic protein (GFAP; dilution 1 : 200; BD Bioscience, Franklin Lakes, NJ) was used as the primary antibody, and Alexa Fluor 488 (Molecular Probes Inc., Eugene, OR, USA) as the secondary antibody. The *μ*-opioid receptor (MOR; generated and gifted by Dr. Masahiko Watanabe, Hokkaido University, Sapporo, Japan), [39, 40] hydroxytryptamine transporter (HTT; generated and gifted from Dr. Masahiko Watanabe, Hokkaido University, Sapporo, Japan), [41] and calcitonin gene-related peptide (CGRP; dilution, 1 : 8000; Sigma C8198), followed by Alexa Fluor 488, were used to evaluate sensory-related synapses and neurons. The areas of HTT-, MOR-, and CGRP-positive cells in the spinal dorsal horn at the level of 10 mm caudal from the injured epicenter were semiautomatically analyzed from transverse sections [42]. Transplanted GFP-positive BMSCs were examined by immunofluorescent staining using a primary rabbit monoclonal anti-GFP antibody (dilution, 1 : 75; 2956S; Cell Signaling Technology, Japan) and confirmed by double fluorescence immunohistochemistry [26]. Antibody for the neuronal nuclear antigen (NeuN, dilution 1 : 100; MAB377, Millipore, Billerica, MA, USA) was used to determine the transdifferentiation of transplanted GFP-positive cells around the peridamaged area. A total of five nonoverlapping lesions on the rostral and caudal side of the damaged area were randomly selected, and positive cells were semiquantitatively counted. The number of the GFP-positive cells, as well as the ratio of double positives for NeuN and GFP, was calculated as previously described [28].

### 2.10. Quantification of Dorsal Corticospinal Tract

For evaluating the dorsal corticospinal tract (dCST), animals (*n* = 3 each) at 49 days after the SCI were used as previously described [31, 32]. The rats were anesthetized, and the midline incision wound was reopened. A 10 *μ*l Hamilton syringe needle was inserted 10 mm rostral from the center of injury at the depth of 1.5 mm into the spinal cord. Fluoro-Ruby (FR; 2 *μ*l; 10,000 molecular weight, D-1817; Molecular Probes, Eugene, OR) was used as a fluorescent axonal tracer and was injected using an automatic microinjection pump into the spinal cord 0.5 mm lateral from the midline on both sides of spinal cord over 3 min. The needle was kept in the spot for 3 min and then slowly withdrawn to minimize the tracer leakage. After closing the skin, the animals were returned to the cages. Five days later, the rats were sacrificed for the visualization of the residual dCST axons. Transverse sections 10 mm caudal from the injured area were examined. The dCST is shown to exist in the ventral one-third of the dorsal funiculus, and the number of FR-labeled axons was bilaterally counted at this point.

### 2.11. Measurement of Hepatocyte Growth Factor (HGF) Secretion Capacity of the Cell Sheet by Enzyme-Linked Immunosorbent Assay (ELISA)

Trophic factor secretion from the cell sheet and stem cells were evaluated to investigate the therapeutic mechanism as previously described with slight modifications [43]. Cell sheets were prepared as previously mentioned, and the nonsheet cells were prepared by simply removing ascorbic acid from the MEM-*α*; cell sheets cannot be prepared without ascorbic acid (*n* = 3). After 18 days of culture, the medium for the cell sheet group was changed to a medium without ascorbic acid, and the cell sheet was cultured for an additional four days. The nonsheet group was prepared with a medium, which does not contain ascorbic acid, for the same duration. After confirming that the cell sheets were successfully produced in the sheet group and not in the nonsheet group, the medium was collected for hepatocyte growth factor (HGF) measurement. Concentrations of HGF (DBB00, R&D Systems, Minneapolis, MN) were measured using an ELISA kit according to the manufacturer's instructions and a spectrophotometer (model 550 reader; Bio-Rad, Hercules, CA). The amount of HGF expressed in the medium was adjusted by the total number of cells in each group.

### 2.12. Statistical Analysis

All data were collected, and all analyses were performed in a blinded manner. The data are presented as means ± standard errors of the means (SEMs). All statistical analyses were performed using JMP Pro 14 (SAS Institute, Inc., Cary NC). The differences between two groups were examined by an unpaired *t*-test or Mann–Whitney *U* test. The differences between three groups were examined using a one-factor analysis of variance (ANOVA) followed by Turkey's HSD test or the Steel–Dwass test. A *p* value of < 0.05 was considered statistically significant.

## 3. Results

### 3.1. BMSC Cell Sheet Improves Functional Motor Recovery and Attenuates Hyperalgesia

**Figure 2:**
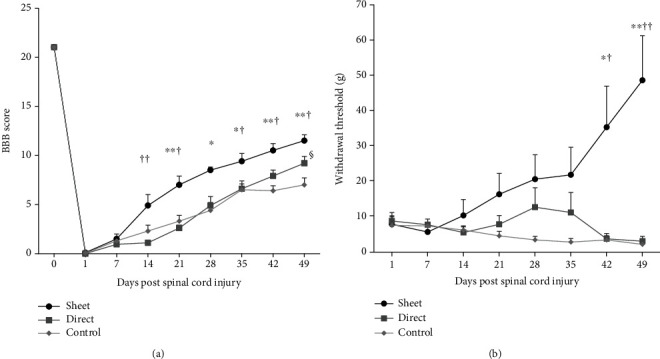
Neurological recovery by cell transplantation. Neurological function was assessed from the Basso, Beattie, and Bresnahan score (a) and the withdrawal threshold determined by von Frey test (b) in the cell sheet group, intramedullary (direct) injection group, and control group (sheet: *n* = 12, direct: *n* = 10, and control: *n* = 10). ^∗^*p* < 0.05, ^∗∗^*p* < 0.01: between the sheet group and control group; ^†^*p* < 0.05, ^††^*p* < 0.01: between the sheet group and direct injection group; and ^§^*p* < 0.05: between the direct injection group and control group.

The cell sheet group showed significant motor functional recovery compared to the intramedullary injection and control groups starting from one week after transplantation, whereas the intramedullary injection group did not show a significant recovery compared to the control group until 6 weeks after transplantation (Figure 2(a)). Furthermore, the cell sheet group showed significant improvement in the neuropathic pain threshold (hyperalgesia), starting 4 weeks after transplantation (Figure 2(b)). Conversely, the intramedullary injection group did not show any improvement compared to the control group.

### 3.2. Cell Sheet Reduces the SCI Area

**Figure 3:**
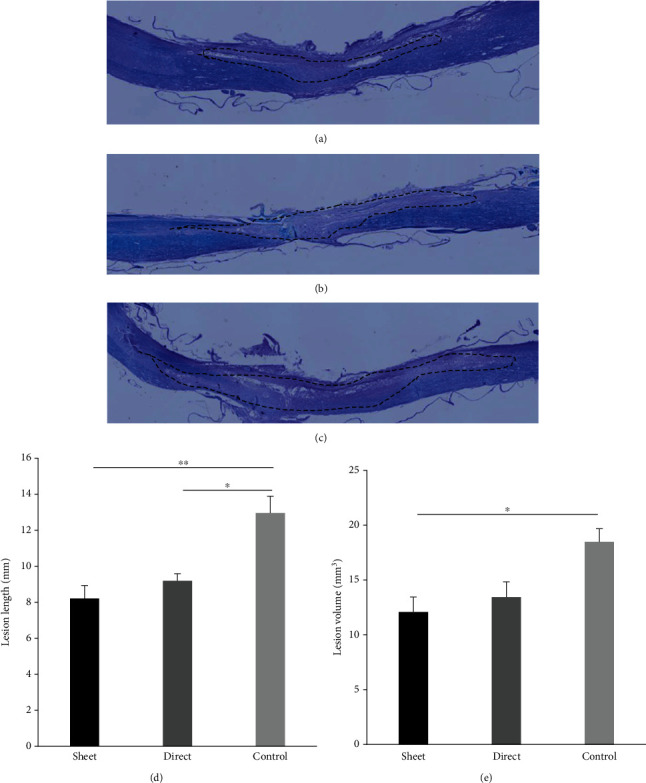
Lengths and volumes of SCI. Length and volumes of SCI were compared between the groups ((a) cell sheet group, (b) intramedullary (direct) injection group, and (c) control group). The BMSC cell sheet significantly reduces the demyelination in lesion length and volume compared to the intramedullary injection group and control group, while the intramedullary injection group shows reduced length compared to the control group (d). The BMSC cell sheet also significantly reduces demyelination lesion volume compared to the control group (e). ^∗^*p* < 0.05, ^∗∗^*p* < 0.01.

Compared to the control group, the cell sheet group showed a significant reduction in injury lesion length (Figures 3(a)–3(d)) and lesion volume (Figure 3(e)) determined by the discontinuation of LFB staining 7 weeks from the insult (*p* < 0.01). On the other hand, the intramedullary injection group showed a statistically smaller lesion length but no difference in the lesion volume compared to the control group.

### 3.3. Cell Sheet Attenuates Activation and Infiltration of Inflammatory Cells around the Injured Area

**Figure 4:**
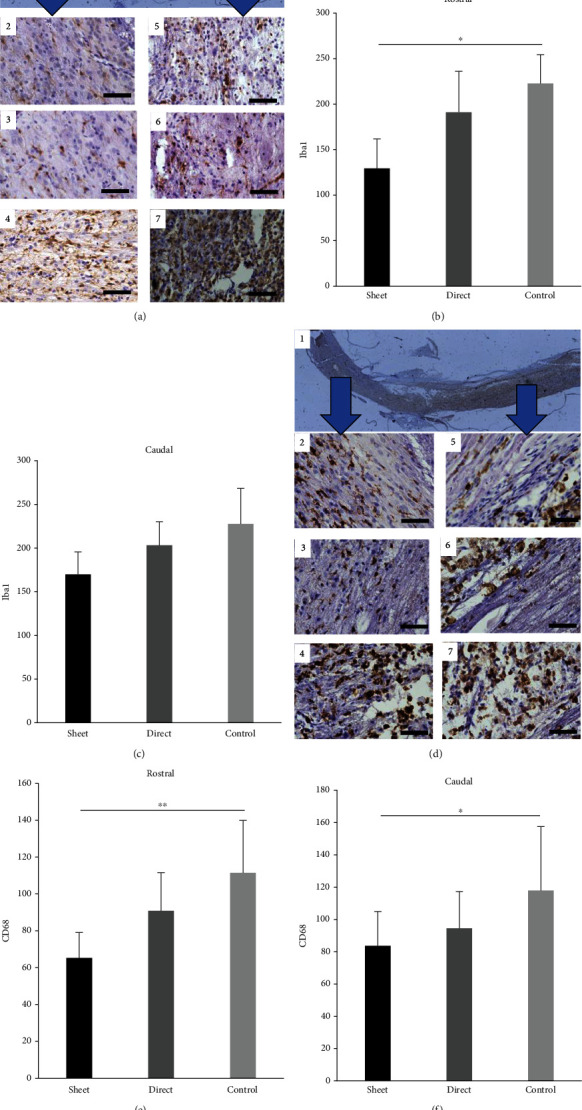
Effect of cell therapy on inflammatory cells. (a–c) Iba1 staining (activated microglia) of the spinal cord 7 weeks after SCI was evaluated on the rostral and caudal sides of the lesion from the epicenter of SCI (a, 1). Iba1 staining is significantly reduced on the rostral side in the cell sheet group (a, 2), compared to the control group (a, 4), while no difference is found compared to the intramedullary (direct) group (a, 3; b). The same trend is seen in the caudal lesion ((a, 5) the cell sheet group, (a, 6) the intramedullary injection group, and (a, 7) the control group; (c)). (d, e) CD68 staining (infiltrated macrophages) of the spinal cord 7 weeks after SCI evaluated on the rostral and caudal sides of the lesion from the epicenter of SCI (d, 1). Iba1 staining is significantly reduced on the rostral side of the cell sheet group (d, 2), compared to the control group (d, 4), while no difference is found compared to the intramedullary (direct) group (d, 3) (e). The cell sheet group also shows significantly fewer CD68-positive cells on the caudal side of the lesion ((d, 5) the cell sheet group, (d, 6) the intramedullary injection group, and (d, 7) control group; f). ^∗^*p* < 0.05, ^∗∗^*P* < 0.01. Scale bar represents 50 *μ*m.

Activated microglia identified by Iba1 staining 7 weeks after SCI were examined at the rostral and caudal portions of the SCI epicenter (Figure 4(a)). The number of activated microglia at the rostral side was significantly lower in the cell sheet group compared with the control group (*p* < 0.05), whereas the direct injection group displayed fewer activated microglia compared to the control group but did not reach statistical significance (Figure 4(a), 2–4 and 4(b)). Although not statistically significant, the cell sheet group displayed fewer activated microglia compared to the direct and control groups on the caudal side of the SCI (Figure 4(a), 5–7 and 4(c)). Similar results were also observed with infiltrated macrophages detected by CD68 staining, in which the cell sheet group showed significantly decreased expression of CD68-positive macrophages compared to the control group on both sides of the epicenter of the lesion, while there was no difference detected in the intramedullary injection group (Figures 4(d)–4(f)).

### 3.4. Cell Sheet Inhibits Glial Scar Formation and Preserved Axon Fibers

**Figure 5:**
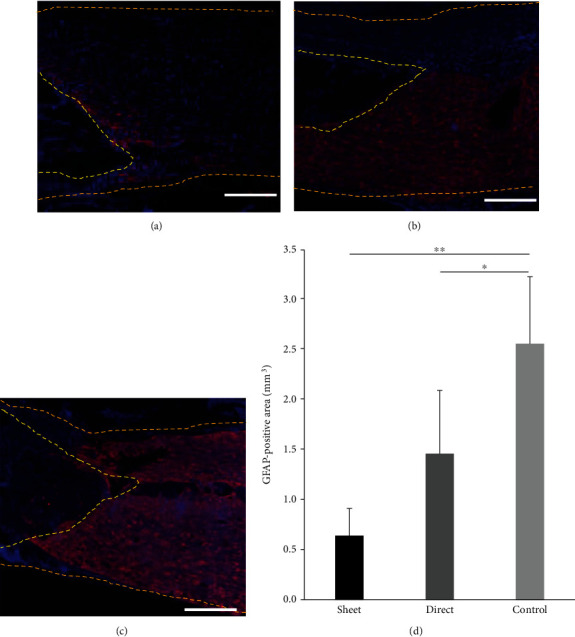
Measurement of glial scar formation. GFAP with DAPI staining on sagittal sections was performed to evaluate the glial scar formation. The left edge of the images (yellow dotted line) is the epicenter of the lesion from the spinal cord injury. The cell sheet group (a) and intramedullary (direct) injection group (b) show significantly less gliosis compared to the control group (c). The cell sheet group and direct injection groups have significantly reduced GFAP-positive areas (d). ^∗^*p* < 0.05, ^∗∗^*p* < 0.01. Scale bar represents 1 mm.

**Figure 6:**
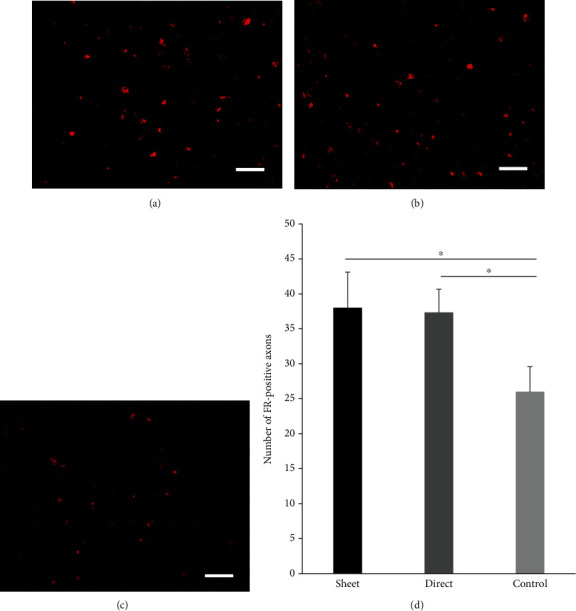
Evaluation of motor fibers in the dorsal corticospinal tract (dCST). The residual/regenerated motor fibers seen by fluor-ruby (FR) dye were evaluated in the dorsal corticospinal tract. The cell sheet group (a) and intramedullary injection (direct) group (b) show significantly more fibers compared to the control group (c). The number of FR-labeled axons in dCST is predominant in the cell sheet group (d). ^∗^*p* < 0.05. Scale bar represents 50 *μ*m.

Glial scars were examined with GFAP staining. The cell sheet group (Figure 5(a)) showed a significant decrease in the GFAP-positive scar area 4 mm caudal of the lesion epicenter and the intramedullary injection group (Figure 5(b)) also showed a decrease in glial scar compared to the control group (Figure 5(c); *p* < 0.01 for the cell sheet group; *p* < 0.05 for the intramedullary injection group). Residual or regenerated axons were analyzed with FR dye staining. The dye, injected into the rostral side of the damaged spinal cord, can be found on the caudal side of the injured spinal cord enabling one to monitor the residual or regenerated axon that delivered the dye through the damaged lesion. The number of FR-labeled axons within the ventral one-third of the dorsal funiculus in the dCST on the caudal side was significantly increased in both the cell sheet and intramedullary injection groups compared to the control group, indicating that stem cells can preserve or regenerate spinal axons within the damaged lesion (Figure 6(d)).

### 3.5. Cell Sheet Preserves the Descending Pain Suppression System and Local Sensory Nerve

**Figure 7:**
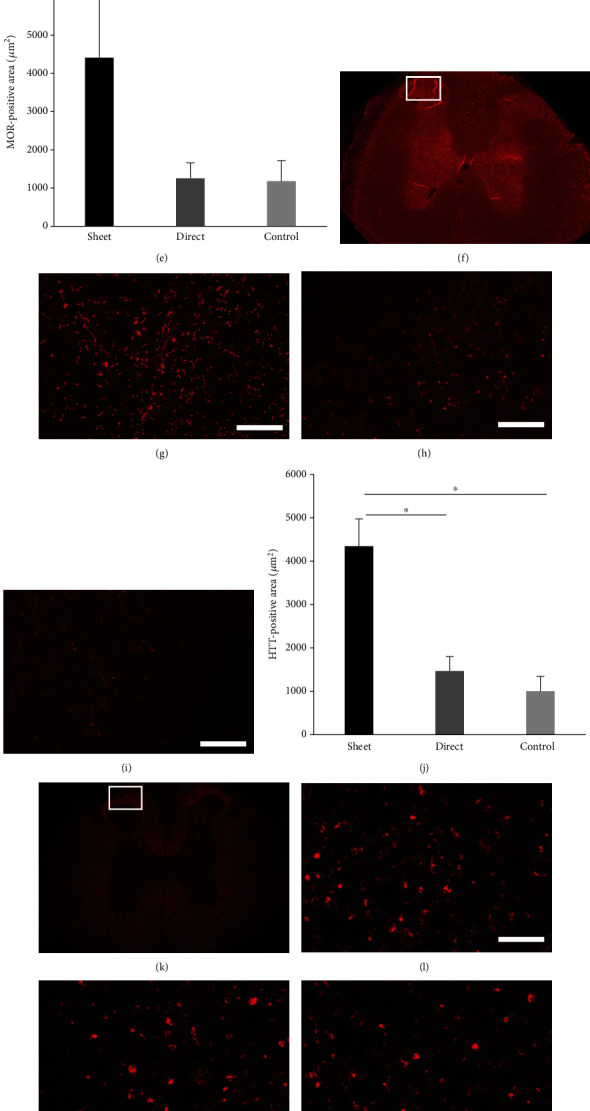
Evaluation of sensory related neuronal networks. (a) Expression of MOR (*μ*-opioid receptor, interneuron) is significantly preserved in the cell sheet group (b) compared with the intramedullary (direct) injection (c) and control groups (d, e). (f) Expression of HTT (hydroxytryptamine transporter, descending pain inhibitory pathway) is also preserved in the cell sheet group (g) compared to the intramedullary (direct) injection (h) and control groups (i, j). (k) However, there is no difference in the staining of CGRP (calcitonin gene-related peptide, peripheral C fiber) among the groups ((l) cell sheet group, (m) intramedullary injection group, and (n) control group) (o). Scale bar represents 20 *μ*m.

Sensory-related nerves were evaluated using multiple staining. Cell sheets preserved the expression of MOR, indicative of local sensory nerves, in the dorsal horn of the spinal cord compared to the intramedullary injection and control groups (Figures 7(a)–7(e)), as well as the expression of the descending pain suppression system as measured by HTT staining (Figures 7(f)–7(j)). On the other hand, there was no significant difference in the expression of peripheral input nerves, CGRP, among the groups (Figures 7(k)–7(o)).

### 3.6. Transplanted Cells Are Found in the Spinal Cord and Show Neuronal Differentiation

**Figure 8:**
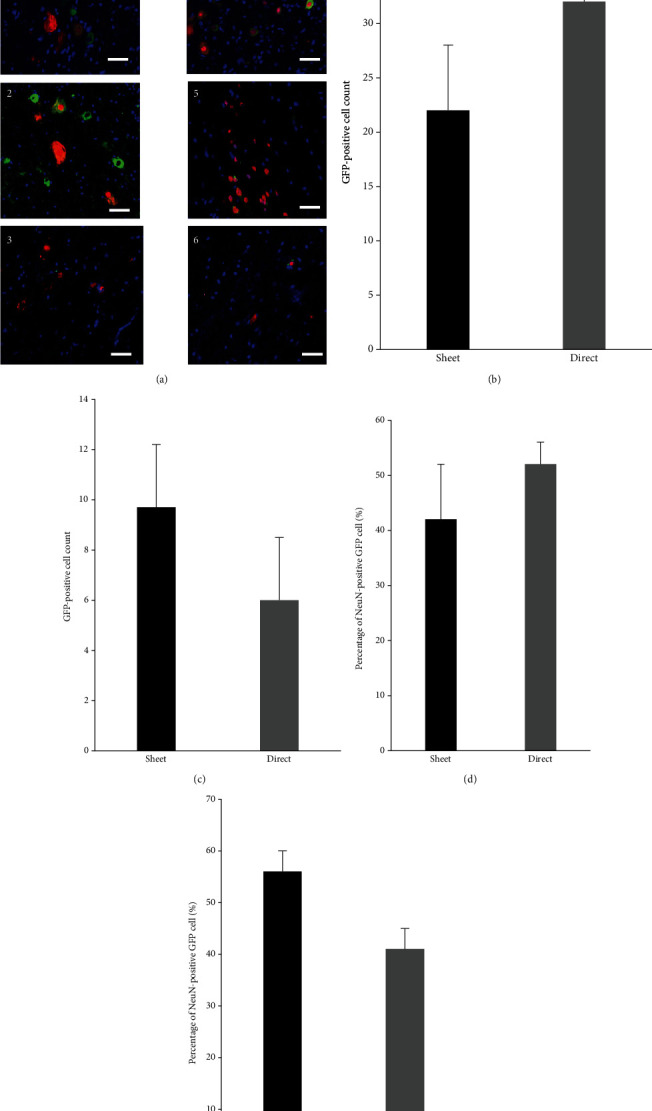
Neuronal differentiation of transplanted cells. (a) GFP (green) and NeuN (red) immunofluorescent staining was performed to confirm the engraftment of transplanted cells inside the spinal cord 5 mm rostral to, or 5 mm caudal to, the epicenter. ((a, 1, 4) caudal and rostral side of the sheet group, respectively; (a, 2, 5) caudal and rostral side of the intramedullary (direct) injection group, respectively; and (a, 3, 6) caudal and rostral side of the control group, respectively). Scale bar represents 50 *μ*m. No differences are observed in the number of GFP-positive cells transplanted by cell sheet or direct injection on the caudal side (b) and rostral side (c). Differentiation of transplanted cells was evaluated with a different transplantation method. The ratio of neuronal differentiation is mostly similar between the cell sheet group and direct injection groups ((d) caudal side, (e) rostral side).

Histological evaluation revealed that transplanted cells from both the cell sheet and intramedullary injection groups were found in the caudal and rostral sides of the SCI lesion (Figures 8(a)–8(c)), whereas no GFP-positive cells were found in the control group. Furthermore, approximately 40–50% of the transplanted cells from both groups were positive for the neuronal marker (NeuN) (Figures 8(d) and 8(e)).

### 3.7. BMSCs in Cell Sheets Show Higher HGF Secretory Capacity than the Same Number of Nonsheet BMSCs

**Figure 9:**
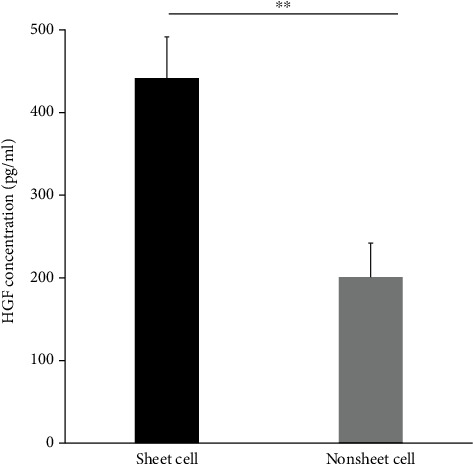
Expression of neurotrophic factors. The expression of hepatocyte growth factor (HGF) was quantitatively examined using an enzyme-linked immunosorbent assay. The cell sheet group releases significantly more trophic factors compared to the noncell sheet group. ^∗∗^*p* < 0.01.

The functional difference between cell sheet and nonsheet cells was further examined by the expression capacity of the trophic factor, HGF. The concentration of HGF was examined using ELISA, and the cells in the cell sheet group released significantly more HGF compared to the same number of nonsheet cells (Figure 9).

## 4. Discussion

Stem cell therapy is believed to be capable of ameliorating or even reversing the damage produced by SCI. [36] Although numerous clinical trials adopting intramedullary or intravenous injection methods have been examined with somewhat satisfactory results [44, 45], the most effective and least invasive treatment method is yet to be determined. In this study, we were able to demonstrate that the subdural administration of a BMSC cell sheet is superior to an intramedullary injection of BMSCs, recovering not only motor functions but also alleviating sensory disturbances induced by SCI. Furthermore, the cell sheet was able to decrease local inflammation and lesion volume and preserve neuronal axons and networks. The sheet appears to also express higher amounts of trophic factors, and the cells were able to migrate into the damaged spinal cord and achieve neuronal phenotypes.

Neuropathic pain (hyperalgesia/allodynia) after SCI is a severe sequela which affects patients' life by mobility impairment and mental depression. Although the precise mechanisms underlying neuropathic pain are not fully elucidated, excessive inflammatory mediators, ion channel dysfunctions, and disruption of descending antinociceptive serotonergic tract are considered to be involved in abnormalities of afferent nerve sensitization [42, 46]. We have previously reported that immunosuppressant FTY720 ameliorate neuropathic pain caused by SCI [31], but the extent of pain relief was much stronger when a cell sheet was applied. Furthermore, this relief was not achieved in the intramedullary injection group. Given that FTY720 has been shown to decrease the accumulation of inflammatory cells at and around the spinal cord, an effect that was observed in both the cell sheet and intramedullary groups, it is likely that the superior recovery rate seen in the cell sheet group is mediated by a different mechanism(s). In addition to the immune-suppression mechanisms, stem cells are known to release various kinds of cytokines and exosomes that protect damaged cells or remyelinate neuronal fibers [36, 47]. These trophic factors may rescue the local environment and repair the abnormal afferent pathway. This is evidenced by the fact that local (MOR) and descendent (HTT) pain suppression neurons, as well as the network as a whole, were improved with cell sheet transplantation, whereas peripheral C fibers were not. Given that the pain pathways are located in the dorsal horn of the spinal cord, implantation of the cell sheet in this location provides a therapeutic response with minimal invasiveness. Besides, two factors may explain the superiority of cell sheets over cell suspensions in reducing neuropathic pain. First, the cell sheet was able to produce higher amounts of HGF, a trophic factor critical for spinal cord recovery, [48] compared to the cell suspension. Many trophic factors have been reported to show similar secretion patterns, and therefore, the cell sheet secretes other factors, including BDNF, TGF-b1, and exosomes, in high amounts as well to ameliorate the inflammation [43]. The higher trophic factor secretion by the cell sheet can be attributed to cell stability; MSCs stabilized by attaching to the substrate through integrins help prevent cell apoptosis, so-called anoikis [49]. The higher amounts of trophic factors inhibited local inflammation. Second, administration of the cell sheet avoids direct spinal cord damage, as the injection itself can cause a certain degree of mechanical injury. Many reports have shown needle tract damages and cell engrafted local injuries in animal models of intraparenchymal transplantation in the brain [50, 51]. Avoiding additional damage to the spinal cord is crucial because the size of the spinal cord is limited compared to that of the brain. Suppression of pain promoted the active voluntary movement of the hind limbs, thereby improving motor function.

We also found that, similar to the intramedullary injection group, the stem cells engrafted by cell sheets were able to migrate into the damaged spinal cord and differentiate into the appropriate neuronal phenotype. This suggests that cell sheets are as effective as intramedullary injections for delivering cells into damaged neuronal tissues. This finding is in line with previous reports demonstrating that cell sheets possess a high migration capacity into neuronal tissues [28, 50], and the engrafted cells contribute to axonal regeneration. Indeed, in our study, both the cell sheet and intramedullary injection groups showed higher numbers of axons in the dCST. In addition to direct neuronal transformation, stem cells may also support local neurogenesis. Quiescent neuronal stem cells have been reported to localize around the central canal of the spinal cord, and stem cells are known to reactivate their proliferation [52]. However, the precise mechanisms of recovery require further examination because the same level of cell engraftment occurs between the cell sheet and direct injection groups, but the recovery was better in the cell sheet group. This may be attributed to trophic factors being more susceptive to recovery from the damage.

Interestingly, we found that the cell sheet released higher amounts of the trophic factor HGF compared to the nonsheet cells. HGF is reported to be one of the most important neurotrophic factors responsible for neuronal recovery in SCI [48]. Our results were in line with a recent report from Bou-Ghannam et al. showing that cell sheets produced from the same material produced higher amounts of trophic factors and anti-inflammatory cytokines, including HGF, vascular endothelial growth factor, and interleukin-10, compared to noncell sheet monolayers [53]. BMSC is an adherent cell that requires attachment to the surrounding surface, and cell sheets enabled these cells to strongly anchor, likely through integrin connections. However, we obtained our data from *in vitro* experiments, and further examination is required to elucidate the function and mechanisms of activities of trophic factors and anti-inflammatory cytokines in *in vivo* studies.

There are several limitations to this study. First, we did not examine the relationship between cell dose and therapeutic efficacy, which is essential to determine a protocol for use in a clinical trial. Given that the subdural space is limited in rats, it was difficult to slide more cell sheets into this area. Future studies using larger animals are necessary to overcome this limitation. Second, different time courses and testing methods were not fully analyzed. We applied cell sheets to the rats 1 week after SCI, which seemed to be the subacute stage; however, neuropathic pain may occur several months after the damage in humans. Therefore, a chronic model where the pain is assessed at least one month after SCI is needed; this is technically difficult in small animals with severe adhesion. Third, while cell sheets portrayed a strong ability to inhibit neuropathic pain, employing other methods of sensory testing may strengthen our data. However, there are currently no established sensory testing techniques, other than the one we adopted, to monitor neuropathic pain in rodent models. Fourth, the control groups did not completely compare with each other. Herein, we adopted three different groups: subdural administration, intraspinal injection, and sham surgery, a group that mimics subdural administration. However, the cell sheet and control groups did not receive intraspinal injections of saline or medium, and the intraspinal group was not injected with subdural nonlive cell sheets, which can elucidate the effects of intraspinal damage and sheet directly. We have previously reported that the effects of intraspinal injection of the cell suspension were superior to that of the cell medium [32], but the effects of the cell sheet without the use of live cells were impossible to generate.

## 5. Conclusions

In conclusion, BMSC cell sheets were able to ameliorate neuropathic pain and promote motor recovery through multiple mechanisms, including, an anti-inflammatory effect, smaller development of gliosis, and the preservation of pain suppression pathways, compared to intramedullary injection. Taken together, these results suggest that cell sheet transplantation is a favorable cell delivery and treatment option for SCI, especially to diminish neuropathic pain.

## Data Availability

The experimental data used to support the findings of this study are available from the corresponding author upon reasonable request.
